# Integrated Analysis of Necroptosis-Related Genes for Prognosis, Immune Microenvironment Infiltration, and Drug Sensitivity in Colon Cancer

**DOI:** 10.3389/fmed.2022.845271

**Published:** 2022-04-11

**Authors:** Rong He, Meiling Zhang, Lian He, Jiabin Huang, Changfeng Man, Xiaoyan Wang, Yakun Lang, Yu Fan

**Affiliations:** ^1^Cancer Institute, The Affiliated People's Hospital of Jiangsu University, Zhenjiang, China; ^2^Department of Gastroenterology, The Affiliated Suqian First People's Hospital of Nanjing Medical University, Suqian, China

**Keywords:** colon cancer, necroptosis, immunotherapy, tumor immune microenvironment, signature

## Abstract

**Background:**

Necroptosis, is intimately linked to tumor development and prognosis and has been considered as a target for anticancer therapy. However, the role of necroptosis-related genes (NRGs) in colon cancer is unclear.

**Methods:**

In the present study, we screened 76 NRGs from previous studies and described the landscape of transcriptomic and genetic variation of NRGs in colon cancer (CC) patient samples. Molecular subtypes of necroptosis in colon cancer were identified by clustering analysis, and these molecular subtypes were linked to patient prognosis and TME cell infiltration characteristics. Then, the NRS-score for predicting overall survival (OS) was built based on the TCGA database and validated in the GSE39582 cohort for its predictive power in CC patients. Besides, the ESTIMATE and CIBERSORT algorithms were applied to explore the relationship between NRS-score and tumor immune microenvironment.

**Results:**

We identified two molecular subtypes associated with necroptosis in CC, which have diverse prognosis and immune microenvironment characteristics. Based on the differentially expressed genes between the two molecular subtypes, we further developed a necroptosis risk score signature, referred to as NRS-score. High NRS-score was associated with poor prognosis in CC through immunosuppressive microenvironment and immune escape mechanisms. The nomogram based on NRS-score showed excellent ability to predict prognosis. In addition, NRS-score presented a positive correlation with tumor mutational burden (TMB) and immune checkpoint blockade (ICB) expression and was closely correlated with multiple anticancer agent susceptibility.

**Conclusion:**

This work revealed a close relationship between necroptosis and the prognosis and immune microenvironment of colon cancer. The NRS-score based on the 8-gene signature may be used to predict the sensitivity of immunotherapy and chemotherapy in colon cancer patients, and provides a foundation for future studies targeting necroptosis and its immune microenvironment.

## Introduction

According to global cancer statistics, colon cancer (CC) is the third most common malignancy and the second leading cause of cancer death worldwide (1). Despite recent advances in diagnostic and therapeutic techniques, tumor recurrence and mortality rates for patients with colon cancer remain high, and the 5-year overall survival (OS) rate for metastatic CC is <15% (2). A proportion of CC patients develop resistance to chemotherapy, immunotherapy, or targeted therapy, leading to cancer recurrence, cancer progression, and death (3–5). Patients with CC receiving the same treatment regimen may also have completely different clinical responses and treatment outcomes, often attributed to the complexity and high heterogeneity at the genetic level and genetic regulation (6, 7). In recent years, with the rapid development of genomics, the classification of colon cancer has shifted from the traditional histological classification to molecular classification (8–11). Nowadays, we can develop distinct therapeutic strategies based on different molecular subtypes in clinical practice, such as KRAS and BRAF mutations, microsatellite instability (MSI), HER-2 amplification, or NTRK fusions (12, 13). Despite our increasing understanding of molecular subtypes of CC, the prognosis prediction based on these molecular subtypes is still less satisfactory. Therefore, more other factors related to prognosis need to be considered to stratify patients more precisely and thus guide more effective and personalized treatment plans.

Necroptosis is a genetically regulated form of programmed necrotic cell death that is mainly activated by receptor-interacting protein kinase 1 (RIPK1), with RIPK3 and mixed lineage kinase domain-like pseudokinase (MLKL) mediated (14). As a combination of necrosis and apoptosis, necroptosis has a dual role in cancer. On the one hand, key regulators of the necroptosis pathway act alone or in combination to promote cancer development and metastasis (15); on the other hand, necroptosis can act as a fail-safe mechanism to prevent tumor progression when apoptosis is not induced (16). In addition, recent studies have identified that targeting the necroptosis pathway could be a promising anti-cancer therapy, especially for tumors that are resistant to conventional treatment (17). For example, in Sarad et al.'s study, edelfosine was found to cause rapid necroptosis in apoptosis-resistant glioblastoma cells, and inhibition of RIPK1 with necrostatin-1 can eliminate edelfosine-induced necroptosis and increase the viability of cancer cells (18). Shikonin was reported to exert anti-cancer effects by mediating RIPK1 and RIPK3 expression to induce necroptosis in pancreatic cancer and osteosarcoma (19, 20). These studies indicated that direct or indirect induction of necroptosis may constitute a new approach for cancer therapy. However, the specific role of necroptosis regulators in the prognosis and anti-cancer mechanisms of CC patients remains unclear. Therefore, identifying the molecular characteristics of necroptosis-related genes may help to elucidate the causes of CC heterogeneity.

This study integrated transcriptomic data from 1,172 CC samples from TCGA and GEO databases and collected 76 necroptosis-related regulators from previous studies. We successfully classified colon cancer patients into two necroptosis-related molecular subtypes with diverse clinical outcomes and tumor microenvironment (TME) infiltration characteristics. In addition, the necroptosis score was established to quantify the level of necroptosis in individual tumors, allowing for more individualized and effective anti-cancer treatment strategies.

## Materials and Methods

### Colon Cancer Dataset Sources and Preprocessing

The RNA sequencing data and corresponding clinical profiles of CC patients were acquired from The Cancer Genome Atlas (TCGA, https://cancergenome.nih.gov/) and the Gene Expression Omnibus (GEO, https://www.ncbi.nlm.nih.gov/geo/). Patients with no survival information were excluded from further analysis. A total of 1,172 patients were enrolled for analysis, which consisted of two GEO cohorts (GSE39582 and GSE17536) and the TCGA-CORD cohort. Baseline information for all colon cancer patient datasets is listed in Supplementary Table 1. Somatic mutation data and copy number variation (CNV) data of CC were obtained from the TCGA database and the University of California, Santa Cruz (UCSC) website, respectively. To eliminate batch effects of different cohorts, we initially converted FPKM values of TCGA-CORD to transcripts per kilobase million (TPM) and then generated a meta-cohort of enrolled patients (TCGA-CORD, GSE39582, and GSE17536) by using the “ComBat” algorithm of the “SVA” package.

### Identification of Differentially Expressed NRGs and Mutation Analysis

Seventy-six necroptosis-related genes (NRGs) were obtained from previous studies (details are presented in Supplementary Table 2). Somatic mutation plots of NRGs in CC patients were generated with the “maftools” R package. The “Circos” R package was used to draw necroptosis regulators for CNV alterations and with the chromosomal location. Differentially expressed NRGs (DENRGs) in tumor and normal tissues in the TCGA-CORD cohort were identified using the “limma” package, with *p* < 0.05. Annotation of DENRGs enrichment were accomplished via Metascape (http://metascape.org).

### Consensus Clustering

We performed consensus clustering using the R package “ConsensusClusterPlus” to identify distinct molecular subtypes associated with necroptosis based on the expression of DENRGs (21). Kaplan-Meier (KM) curves were presented by the R packages “survival” and “survminer.” The GSVA enrichment analysis was performed with the “GSVA” R package (22). The gene set “c2.cp.kegg.v6.2.symbols” was downloaded from the MSigDB database and used to run the GSVA analysis. Adjusted *P* < 0.05 were considered significant. Single sample gene set enrichment analysis (ssGSEA) algorithm was used to quantify the relative abundance of each cell infiltrate in TME of CC. The gene sets used to mark each TME-infiltrating immune cell type were obtained from Charoentong's study (23).

### Differences Analysis Among Molecular Subtypes

The R package “limma” was used to identify DEGs between various subtypes, and the screening criteria were adjusted *P* < 0.05 and | Log2(fold change) | > 0.5 (24). Enrichment analysis based on these differentially expressed genes was performed via the “clusterProfiler” R package (25) for gene ontology (GO) and the Kyoto Encyclopedia of Genes and Genomes (KEGG) (25). Adjusted *P* < 0.05 were considered significant.

### Construction and Validation of Necroptosis-Related Prognostic Signature

The TCGA-CORD cohort contains 433 colon cancer patients and provides the fullest clinical annotation, so we selected it as the training set to construct the prognostic signature. Univariate Cox regression analysis was used to screen out DEGs with significant prognostic value, and then minimum absolute shrinkage and LASSO Cox regression analysis was performed using the R package “glmnet” to further narrow down the candidate genes (26). The NRS-score was calculated using the following equation: NRS-score = Σ (βi × Expi) (β: coefficients, Exp: gene expression level). CC patients in the TCGA cohort were divided into low and high NRS-score subgroups based on the median NRS-score. Kaplan-Meier (KM) curves and receiver operating characteristics (ROC) curves were performed to assess the sensitivity and specificity of the prognostic signature. Patients with colon cancer were divided into groups according to age ( ≤ 65 or >65 years), sex (female or male), T-stage (T1-2 and T3-4), N-stage (N0 and N1-2), and M-stage (M0 and M1). The R package “beeswarm” was used to assess the correlation between the prognostic signature and the above clinical parameters. Univariate COX analysis and multivariate COX analysis were used to examine the prognostic ability of clinical traits including NRS-score. To accurately predict 1, 3, and 5-year OS in CC patients, a prognostic nomogram was created using the “rms” R package based on independent prognostic factors. In addition, the GSE39582 dataset was used as an external validation set to confirm the model's predictive value.

### Tissue Samples

Samples from 6 pairs of CC and adjacent non-tumor tissues were obtained from the Affiliated People's Hospital of Jiangsu University. The study protocol was approved by the Medical Ethics Committee of the Affiliated People's Hospital of Jiangsu University, and written informed consent was obtained from all participants.

### RNA Extraction and Quantitative Real-Time PCR Analysis

According to the manufacturer's protocol, the total RNA was extracted from CC tissues by Trizol reagent (Solarbio, Beijing, China). Subsequently, the extracted RNA was reverse transcribed using the PrimeScript RT kit and gDNA Eraser (TaKaRa, Otsu, Japan). SYBR Green-based quantitative real-time PCR was used for analysis. Data were calculated by the 2–ΔΔC(T) strategy and standardized with GAPDH. The primers used in the real-time PCR analysis are listed in Supplementary Table 3.

### Evaluation of Immune Microenvironmental Characteristics

To investigate the relationship between the NRS-score and TME, the ESTIMATE algorithm was applied to quantify the immune score, stromal score, ESTIMATE score, and tumor purity for each patient in TCGA-CORD (27). The R package “CIBERSORT” was used to estimate the relative proportion of twenty-two types of tumor-infiltrating immune cells (TICs) in each sample (28). To elucidate the potential role of the constructed gene signature in CC immunotherapy, we inspected the differential expression of 47 immune checkpoint genes between the different NRS-score groups (29).

### Evaluation of the Efficacy of Anti-cancer Drugs

Data from the Genomics of Drug Sensitivity in Cancer (GDSC) database were used to predict the sensitivity of CC patients to chemotherapeutic and targeted therapeutic agents. The “pRRophetic” R package was used to estimate the half maximal inhibitory concentration (IC50), which was extensively utilized in medical studies (30, 31).

### Statistical Analysis

All data analyses were performed using R software (version 4.0.5). Wilcoxon test was used to compare the differences in gene expression levels between cancer and normal tissues. Correlation tests were used using the Spearman coefficient test. The statistical significance threshold was considered as *P* < 0.05 if not explicitly mentioned.

## Results

### Landscape of Genetic Variation and Differential Expression of Necroptosis-Related Regulators in CC

**Figure 1:**
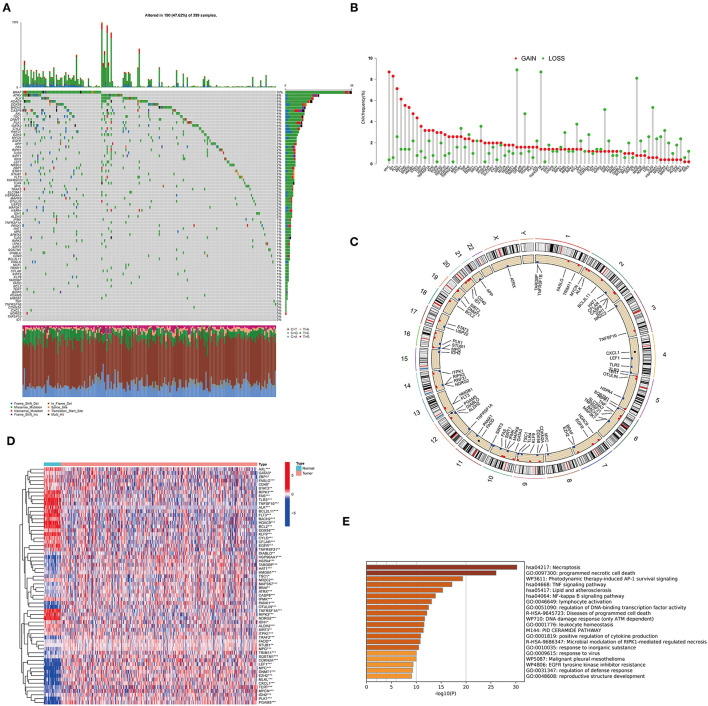
Characteristics and differences of necroptosis-related regulators (NRGs) in CC. **(A)** Mutation landscape of 399 colon cancer patients from the TCGA-CORD cohort. **(B)** The frequency of CNV variation in NRGs is common. Pink dots: amplification frequency; green dots: deletion frequency. **(C)** Location of CNV alterations of NRGs on chromosomes. **(D)** Heatmap of DENRGs expression in the TCGA cohort (*P < 0.05, **P < 0.01, ***P < 0.001). **(E)** Functional annotation of differentially expressed necroptosis-related genes using Metascape.

A total of 76 NRGs were included in the present study. We first explored the incidence of somatic mutations in the 76 NRGs of the TCGA-CORD cohort. The results indicated that 190 (47.62%) of the 399 CC samples showed regulatory mutations associated with necroptosis (Figure 1A). Among them, BRAF (15%) was the mutated gene with the highest frequency, followed by ATRX (8%) and ALK (6%). Since BRAF showed the highest mutation frequency, we evaluated the relationship between BRAF mutation and NRG expression. The results suggested that the expression levels of 37 of 76 PRGs were significantly correlated with BRAF mutation status (Supplementary Figure 1). We also investigated the frequency of CNV alterations and found that all 76 NRGs showed prevalent CNV alterations (Figure 1B). MYC and ID1 exhibited the most prominent copy number amplification, while TARDBP, TNFRSF1B, and TLR3 displayed remarkable copy number deletions. The altered position of the CNVs of NRGs on the chromosomes was illustrated in Figure 1C. To elucidate whether the expression levels of NRGs in colon cancer patients are influenced by these genetic variants, we than identified the expression of NRGs between the cancer and normal tissues in the TCGA-CORD cohort. A total of 62 DENRGs were identified (*P* < 0.05), of which 31 regulators were upregulated and 31 were downregulated in cancer tissues (Figure 1D, Supplementary Table 4). However, there was no significant correlation between the differential expression of these NRGs and CNV alterations. GO enrichment and Metascape analysis revealed that the differential genes were significantly enriched in necroptosis, programmed necrotic cell death, photodynamic therapy-induced AP-1 survival signaling, and TNF signaling pathway (Figure 1E).

### Identification of Necroptosis Subtypes in CC

**Figure 2:**
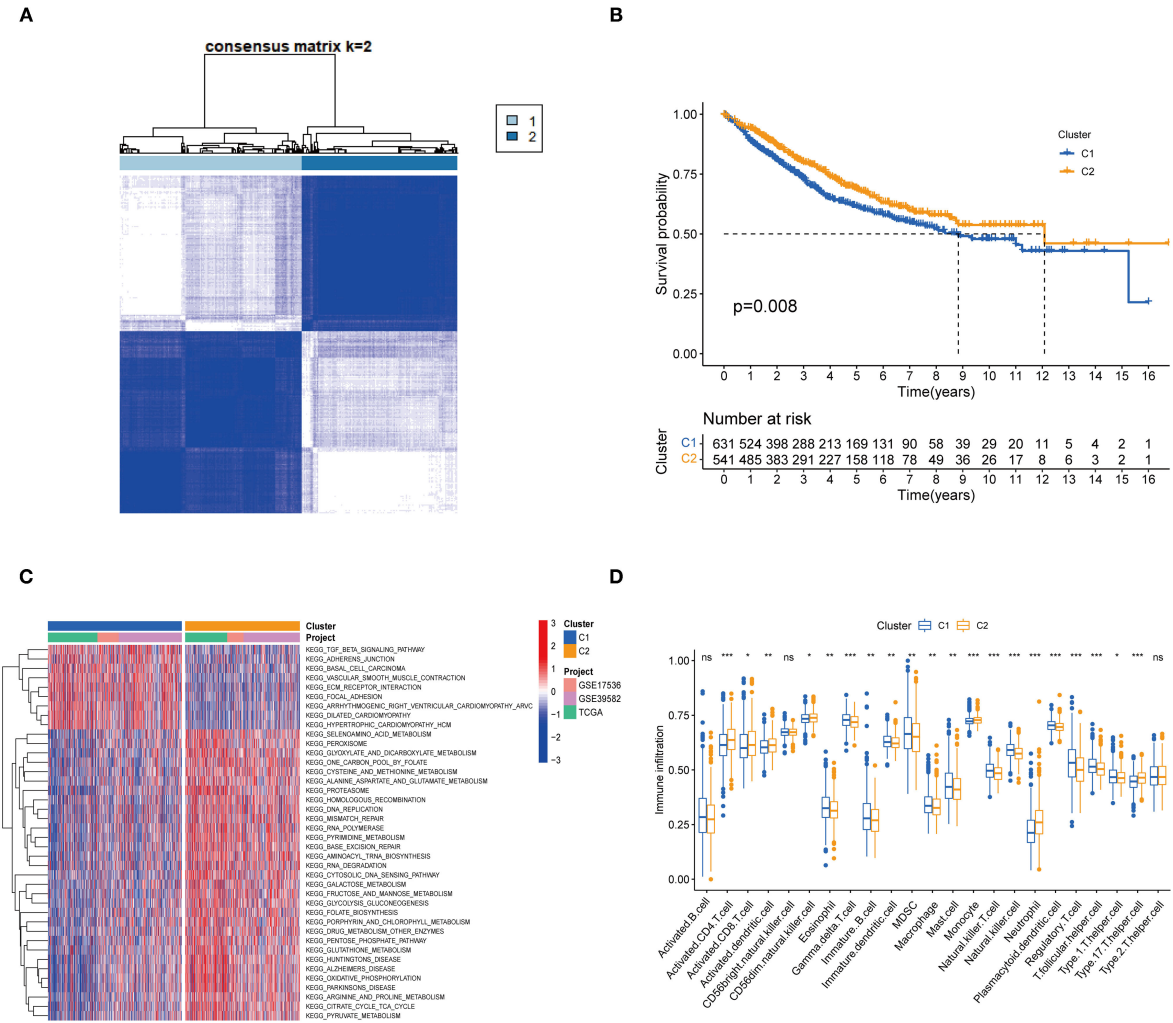
Subgroups of colon cancer related by necroptosis-related regulators. **(A)** Consensus clustering of colon patients for *k* = 2 in the meta-cohort (GSE39582, GSE17536 and TCGA-CORD). **(B)** Kaplan-Meier curves (Log-rank test, *P* = 0.008) for overall survival (OS) of two necroptosis-related molecular subtypes. **(C)** GSVA enrichment analysis showed the activation status of biological pathways in two necroptosis-related molecular subtypes. Red: activation of biological pathways, blue: inhibition of biological pathways. **(D)** ssGSEA analysis showed the difference in the abundance of TME-infiltrated cells between the two subtypes (*P < 0.05; **P < 0.01; ***P < 0.001; ns, not statistically significant).

One thousand one hundred and seventy two colon cancer patients were included in a meta-cohort of GSE39582, GSE17536 and TCGA-CORD. In the meta-cohort, unsupervised clustering was used to classify CC patients based on the expression profiles of 62 DENRGs. Our results showed that *k* = 2 seemed to be the best choice for classifying the whole cohort into molecular subtypes (Figure 2A, Supplementary Table 5), which were named C1 (*n* = 631) and C2 (*n* = 541). Survival analysis revealed a significant survival advantage with the C2 subtype (*p* = 0.008, Figure 2B). To explore the biological differences between these different necroptosis subtypes, we performed GSVA enrichment analysis (Figure 2C, Supplementary Table 6). The results showed that the C1 subtype was mainly enriched in some stromal and oncogenic activation pathways, such as TGF-β signaling pathway, ECM-receptor interaction, and basal cell carcinoma; the C2 subtype was mainly enriched in metabolic and repair-related pathways, such as glyoxylate and dicarboxylate metabolism, mismatch repair, and base excision repair. We then explored whether these two necroptosis subtypes had distinct TME characteristics (Figure 2D). It was found that the most significant immune infiltrating cells in the C2 subtype were activated CD4 T cells, activated CD8 T cells, activated dendritic cells, CD56 bright natural killer cells, monocyte, and neutrophil. And eosinophils, immature B cells, immature dendritic cells, natural killer cells, mast cells, MDSC, macrophage, plasmacytoid dendritic cells, regulatory T cells, and T follicular helper cells in C1 subtypes showed greater infiltration. Previous studies suggested that although some tumor tissues are enriched with abundant immune cells, these immune cells do not penetrate the tumor parenchyma and are only present in the surrounding matrix of tumor cell nests (32). Therefore, we speculated that the poorer prognosis of the C1 subtype might be related to the activation of the matrix in TME.

### Identification of DEGs Associated With Necroptosis Phenotype

**Figure 3:**
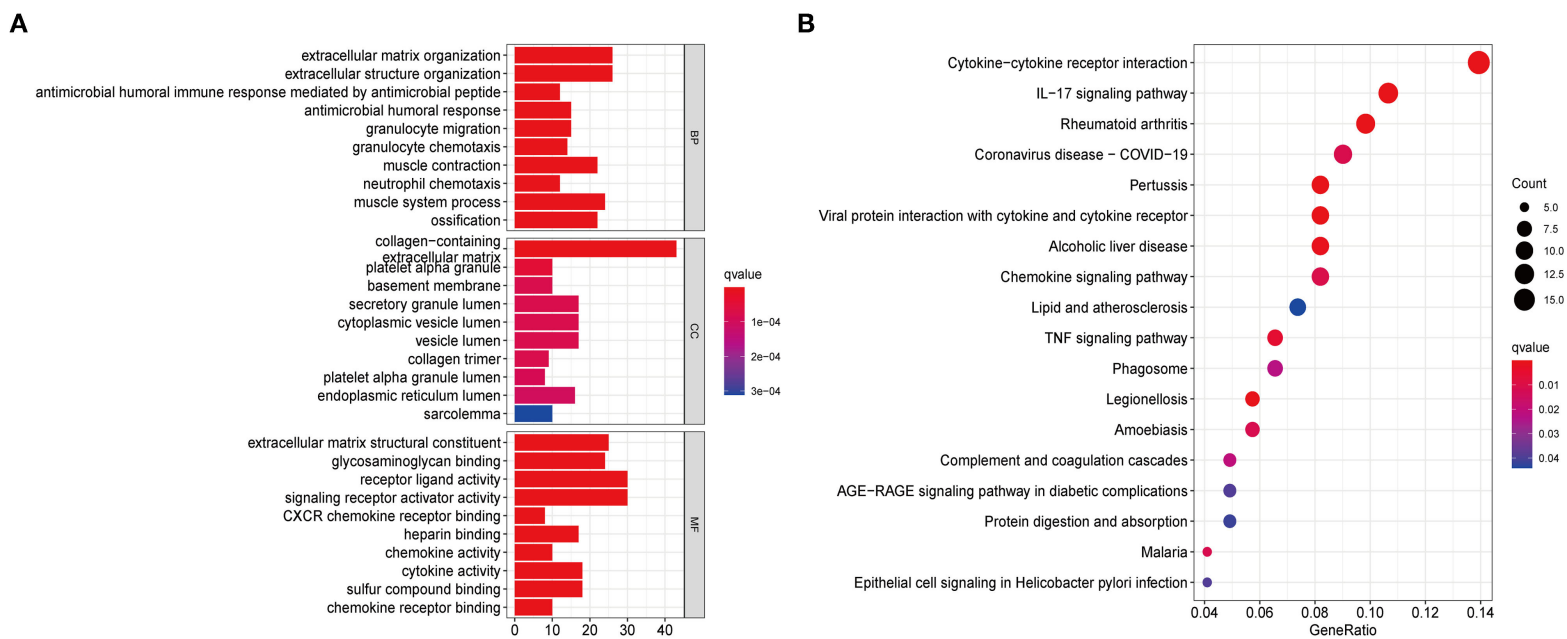
Functional enrichment analysis based on DEGs between distinct necroptosis molecular subtypes. **(A)** GO functional enrichment analysis with bar plot (BP, biological process; CC, cellular component; MF, molecular function). **(B)** KEGG pathway enrichment analysis with bubble plot.

To further explore the functional and pathway differences among necroptosis subtypes, we identified 265 DEGs associated with necroptosis phenotypes using the “limma” R package (Supplementary Table 7). GO enrichment analysis (Figure 3A) showed that these DEGs were significantly enriched in immune function-related terms such as neutrophil chemotaxis, cytokine activity, and chemokine receptor binding. KEGG enrichment analysis (Figure 3B) showed that DEGs were significantly enriched in immune-related pathways, such as Cytokine-cytokine receptor interaction, Chemokine signaling pathway, and IL-17 signaling pathway. These results reconfirmed that necroptosis plays a crucial role in the immune regulation of TME.

### Construction and Validation of a Gene Signature Based on Necroptosis-Related Subtypes

**Figure 4:**
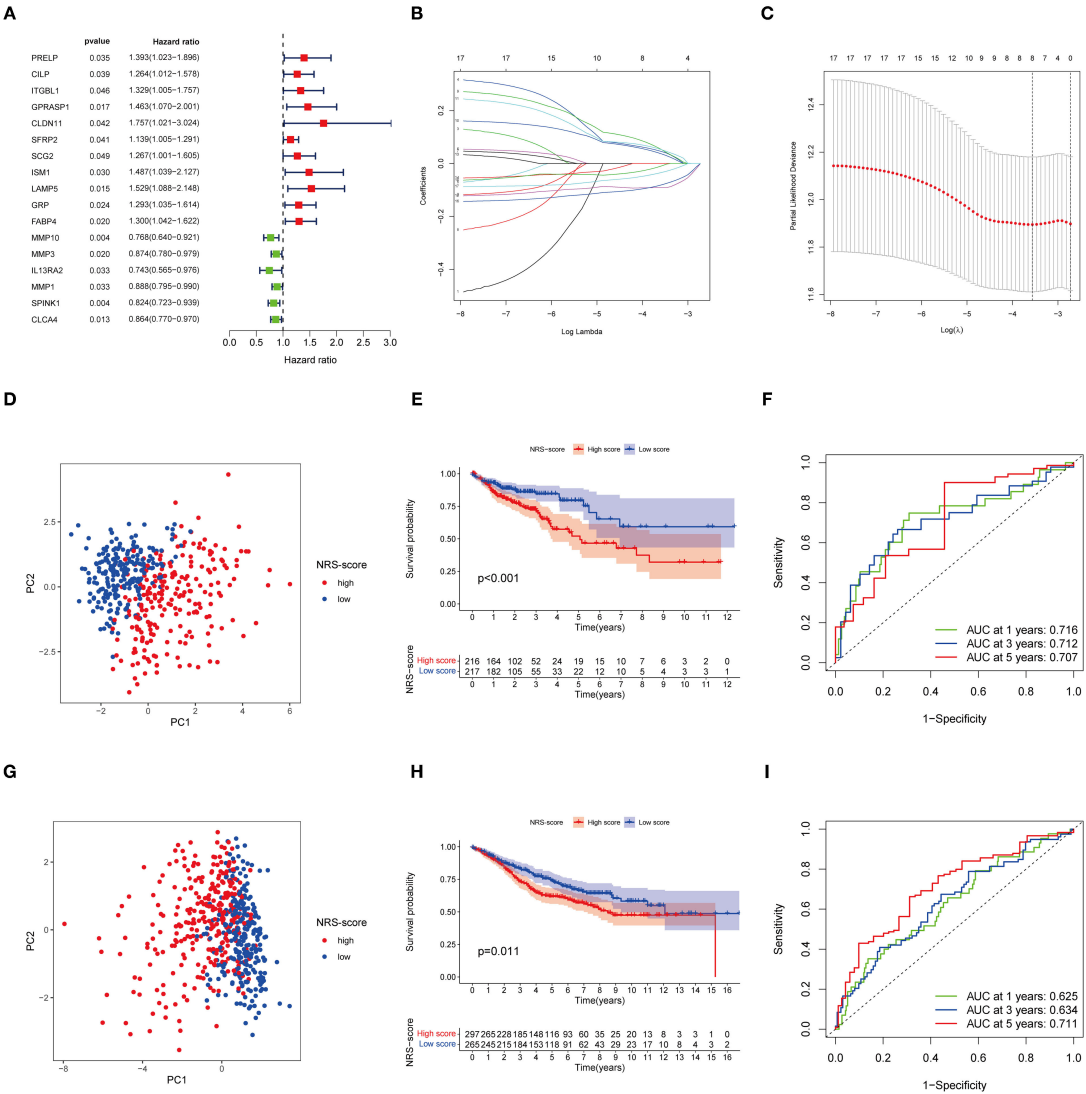
Identification and validation of necroptosis-related prognostic signature in colon cancer. **(A)** Univariate Cox regression analysis showed a total of 17 prognostic-related genes. **(B,C)** The least absolute shrinkage and selection operator (LASSO) algorithm was used to obtain the coefficients of the genes selected to build the prognostic signature. **(D)** The PCA analysis showed that colon cancer patients in the TCGA cohort were significantly divided into high NRS-score and low NRS-score groups. **(E)** Kaplan-Meier curve of OS for patients in the high NRS-score and low NS-score groups from the TCGA cohort. **(F)** Time-dependent ROC curves for predicting overall survival at 1, 3, and 5 years in the TCGA cohort. **(G)** The PCA plot based on the NRS-score in the GSE39582 cohort. **(H)** Kaplan-Meier curve for the high NRS-score and low NRS-score subgroups in the GSE39582 cohort. **(I)** ROC curves for predicting 1-, 3-, and 5-year OS in the GSE39582 cohort.

Although our discoveries identify a role of necroptosis molecular subtypes in prognosis and regulation of immune infiltration, these analyses are based only on patient groups and do not accurately predict necroptosis characteristics in individual tumors. Therefore, we next constructed a prognostic signature based on differential genes of molecular subtypes for application to the diagnosis and treatment of each patient. We first screened 17 genes with prognostic value using univariate Cox regression analysis based on 265 DEGs associated with necroptosis phenotypes with TCGA-CORD as the training set (Figure 4A). And then we used LASSO Cox regression analysis to identify key genes with the best prognostic value by reducing the dimensionality and calculating the correlation coefficients of genes (Figures 4B,C). Finally, eight optimal genes (GPRASP1, LAMP5, GRP, FABP4, MMP10, MMP1, SPINK1, and CLCA4) were selected to construct the prognostic signature (Table 1), which we named as “NRS-score.” The formula is as follows: NRS-score= (0.0444 ^*^ GPRASP1 expression) + (0.0508^*^ LAMP5 expression) + (0.0354 ^*^ GRP expression) + (0.0514^*^ FABP4 expression) + (−0.0910^*^ MMP10 expression) + (−0.0081 ^*^ MMP1 expression) + (−0.0799^*^ SPINK1 expression) + (−0.0332^*^ CLCA4 expression). CC patients in the TCGA cohort were divided into high NRS-score (*n* = 216) and low NRS-score groups (*n* = 217) according to median risk cut-off values. Principal component analysis of the different NRS-score groups showed satisfactory separation (Figure 4D). Kaplan-Meier curves showed a significant survival advantage for patients in the low NRS-score group (*p* < 0.001, Figure 4E). To assess the predictive power of the NRS-score, we created ROC curves for 1, 3, and 5-year survival, with AUC values of 0.716, 0.712, and 0.707, respectively, which indicated well sensitivity and specificity (Figure 4F).

**Table 1:** Correlation coefficients of 8 key genes in necroptosis signature.

**Gene**	**Coef**
GPRASP1	0.044407
LAMP5	0.050843
GRP	0.035409
FABP4	0.051369
MMP10	−0.09095
MMP1	−0.00814
SPINK1	−0.07989
CLCA4	−0.03322

To further verify the robustness of the model, we used the GSE39582 cohort as an external validation set. Based on the median cut-off values of NRS-score in the TCGA cohort, they were divided into a high scoring group (*n* = 297) and low scoring group (*n* = 265) (Figure 4G). The results of the Kaplan-Meier survival analysis remained consistent with the training set, with the high NRS-score group displaying a significantly poorer prognosis than the low NRS-score group (Figure 4H). The ROC curves showed that the model showed promising performance in predicting the OS of CC patients (Figure 4I).

### Clinical Correlation Analysis of the Prognostic Model

**Figure 5:**
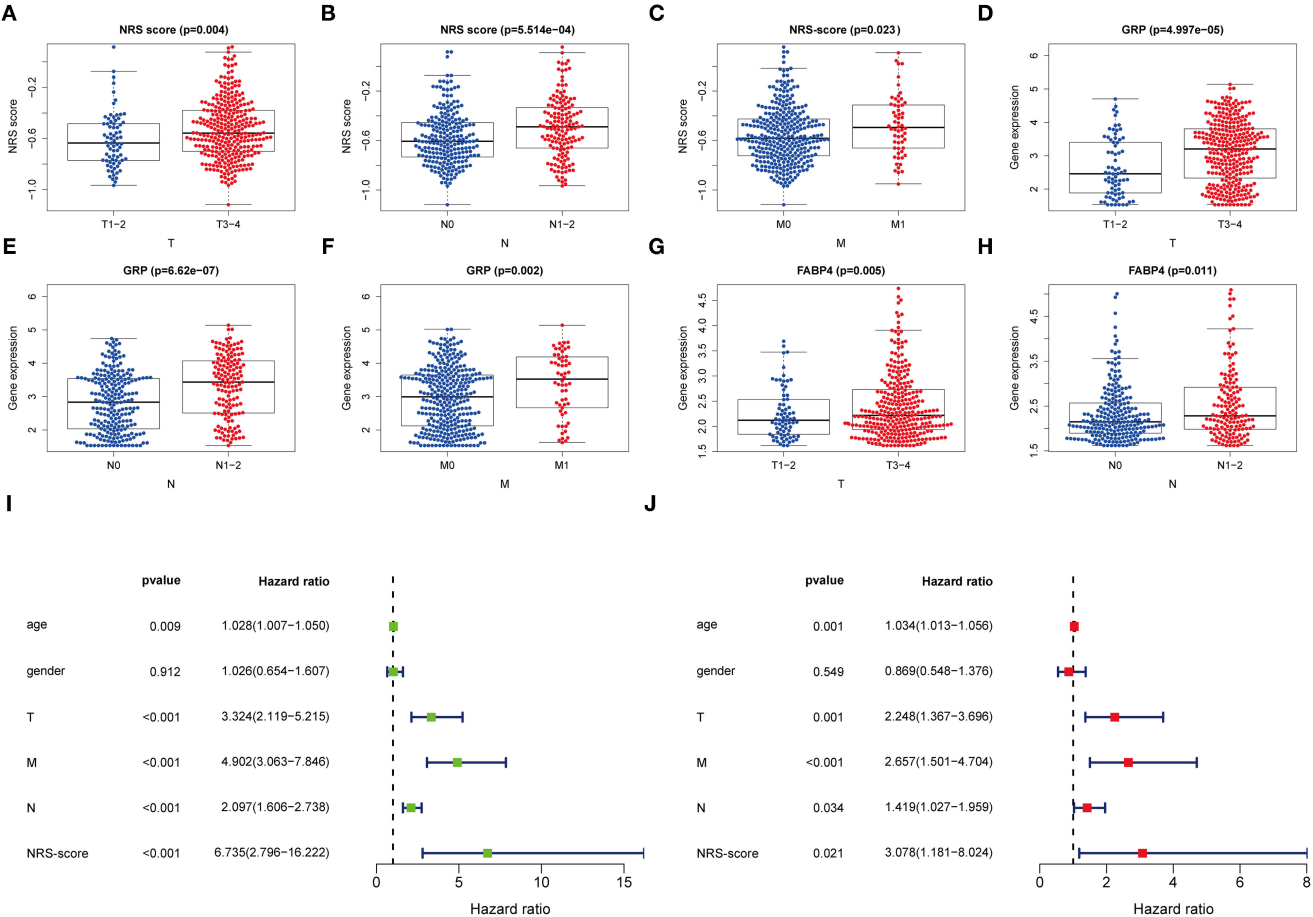
Clinical application value and independent prognosis analysis. **(A–C)** Correlation between NRS-score and **(A)** T-stage, **(B)** N-stage, and **(C)** M-stage. **(D–F)** Correlation between GRP and **(D)** T-stage, **(E)** N-stage, and **(F)** M-stage. **(G,H)** Correlation between FABP4 and **(G)** T-stage, and **(H)** N-stage. **(I)** Univariate COX regression analysis based on NRS-score and clinical characteristics. **(J)** Multivariate COX regression analysis based on NRS-score and clinical characteristics.

For further verifying the significance of the NRS-score in clinical practice, we checked its correlation with the available clinical traits of CC. The results showed that NRS-score was significantly associated with high T-stage, N-stage, and M-stage in patients with colon cancer (Figures 5A–C). In addition, high GRP expression was significantly correlated with high T-stage, N-stage, and M-stage (Figures 5D–F). And high FABP4 expression was significantly correlated with high T-stage and N-stage (Figures 5G,H). All results are shown in Supplementary Table 8. To determine whether the NRS-score could independently predict OS in CC patients, we performed univariate and multivariate Cox regression analyses in the TCGA cohort by incorporating age, gender, TNM stage, and NRS-score. Univariate Cox analysis showed that NRS-score, age, T-stage, N-stage, and M-stage were significantly associated with OS (Figure 5I). Multivariate Cox analysis further confirmed that NRS-score, age, T-stage, N-stage, and M-stage were independent prognostic factors affecting patients with colon cancer (Figure 5J).

### Construction of the Nomogram and Comparison of the Prognostic Signature

**Figure 6:**
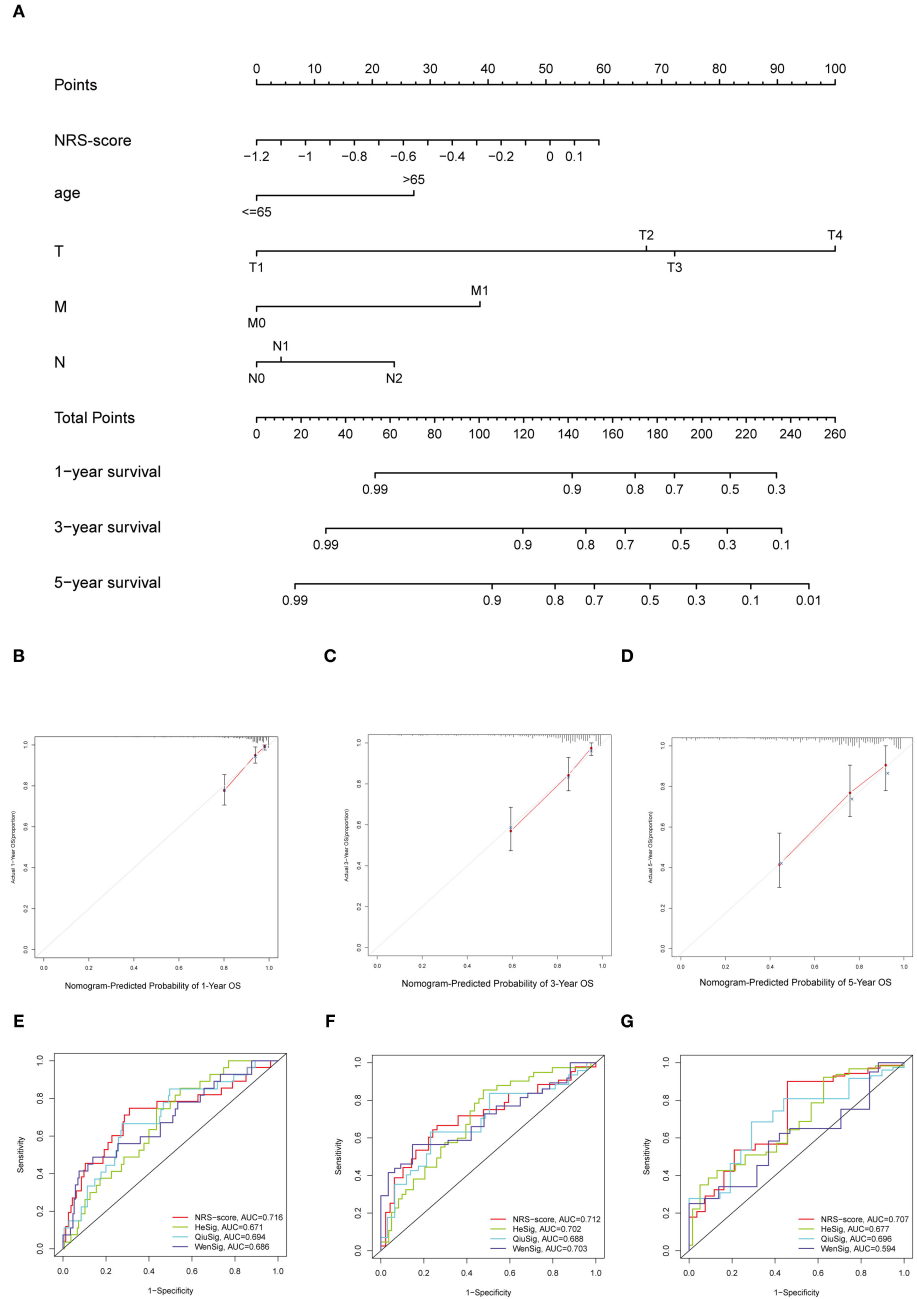
Establishment of the nomogram for predicting overall survival for patients with colon cancer. **(A)** Nomogram based on prognostic signature for predicting 1-, 3-, and 5-year survival in patients in colon cancer. **(B–D)** Calibration curves of nomogram for predicting **(B)** 1-year, **(C)** 3-year, and **(D)** 5-year survival. **(E–G)** Comparison of the area under the time-dependent ROC curves for NRS-score, HeSig, QiuSig, and WenSig at **(E)** 1-, **(F)** 3-, and **(G)** 5-years OS.

To establish a clinically applicable survival prediction method for CC patients, we combined the above five independent prognostic factors to construct a nomogram to predict the 1-, 3-, and 5-year survival probabilities of CC patients (Figure 6A). Then, we analyzed the accuracy of the model by calibration curves. The results showed that the 1-year (Figure 6B), 3-year (Figure 6C), and 5-year (Figure 6D) survival probabilities predicted by the nomogram were closely correlated with the actual observed survival probabilities, confirming the reliability of the nomogram.

In addition, to assess the predictive strength of the NRS-score for clinical use in the CC patient cohort, we compared our prognostic signature with other recently reported CC signatures. Relevant prognostic genes were obtained from the corresponding literature: the hypoxia-related prognostic signature from He (referred to as HeSig), the xenobiotic metabolism-related prognostic signature from Wen (referred to as WenSig), and the inflammation-related prognostic signature from Qiu (referred to as QiuSig). We found that our model performed slightly better than the other three models in predicting CC prognosis (1, 3, and 5-year survival) through the comparative analysis of these models (Figures 6E–G).

### Validation of Prognostic Signature Gene Expression

**Figure 7:**
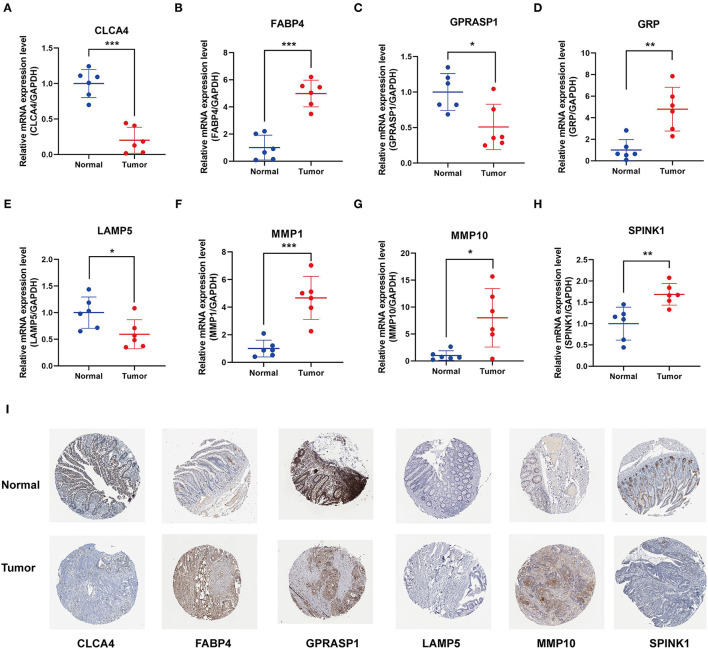
Validation of 8 prognostic signature genes expression. **(A–H)** Differential expression of **(A)** CLCA4, **(B)** FABP4, **(C)** GPRASP1, **(D)** GRP, **(E)** LAMP5, **(F)** MMP1, **(G)** MMP10, and **(H)** SPINK1 in the colon cancer and adjacent tissues (*P < 0.05, **P < 0.01, and ***P < 0.001). **(I)** Representative immunohistochemical staining of signature genes in the Human Protein Atlas (HPA).

We examined the mRNA levels of eight signature genes in six pairs of colon cancer tissues and adjacent normal tissue samples collected from our hospital. The results showed that, compared with the corresponding normal tissues, the expression levels of MMP10, GRP, MMP1, FABP4, and SPINK1 were elevated in CC tissues, whereas the expression levels of GPRASP1, CLCA4, and LAMP5 were downregulated (Figures 7A–H). Interestingly, the FABP4 expression detected in the qRT-PCR assay was not consistent with the results of the Gene Expression Profiling Interactive Analysis (GEPIA) analysis (Supplementary Figure 2). In another study, FABP4 was similarly highly expressed in colon cancer tissues. Therefore, further research into the role of FABP4 in colon cancer is needed. Immunohistochemical (IHC) staining of protein levels was downloaded from the Human Protein Atlas database (33) (Figure 7I). SPINK1, FABP4, and MMP10 showed darker staining in tumor tissues, and GPRASP1, CLCA4, and LAMP5 showed lighter staining. However, GRP and MMP1 were not detected.

### Immune Microenvironment Characteristics in Various NRS-Score Groups

**Figure 8:**
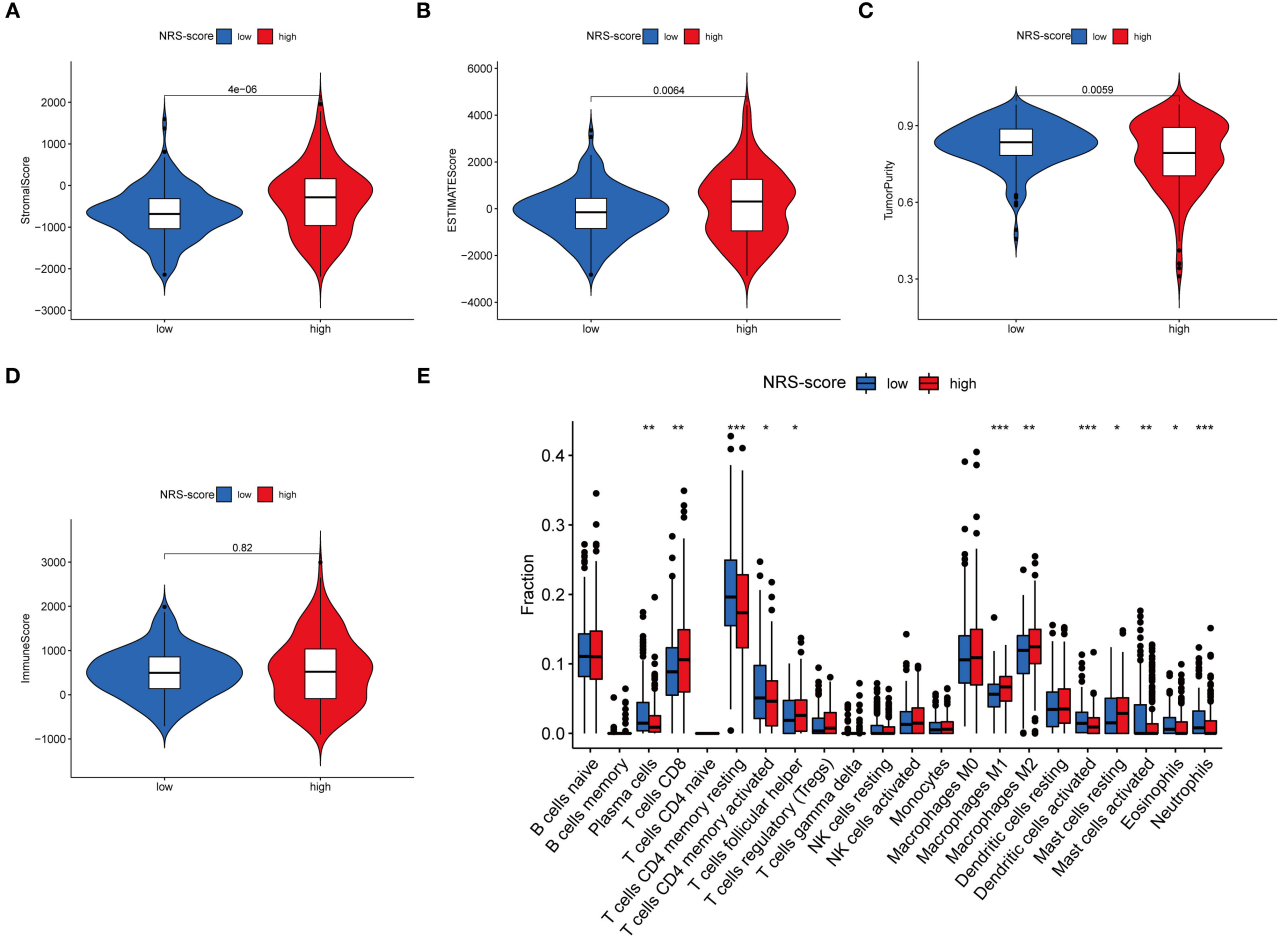
Immune microenvironment infiltration characterization of the prognostic signature. **(A)** Stromal score between high NRS-score and low NRS-score groups. **(B)** ESTIMATE score between high NRS-score and low NRS-score groups. **(C)** Tumor purity between high NRS-score and low NRS-score groups. **(D)** Immune score between the high NRS-score and low NRS-score groups. **(E)** Differences in the abundance of 22 immune cells infiltrating CC tumor samples in the low NRS-score and high NRS-score group (*P < 0.05, **P < 0.01, and ***P < 0.001).

Since TME plays a crucial role in cancer development and treatment, we further explored the differences in immune characteristics between the various NRS-score groups. ESTIMATE analysis showed that both the stromal score (*p* < 0.001, Figure 8A) and ESTIMATE score (*p* = 0.0064, Figure 8B) were significantly higher in the high NRS-score group than in the low NRS-score group, while the tumor purity in the high NRS-score group (*p* = 0.0059, Figure 8C) was significantly lower than that in the low NRS-score group, and no significant difference was seen in the immune score (Figure 8D). We further analyzed the proportion of tumor-infiltrating immune subgroups using the CIBERSORT algorithm and constructed 22 immune cell profiles in the CC samples. Combining the results of correlation analysis (Supplementary Figure 3) and difference analysis (Figure 8E), a total of 12 TICs were significantly correlated with the NRS-score. Among them, T cells follicular helper, T cells CD8, mast cells resting, macrophages M1, and macrophages M2 were positively correlated with NRS-score, while T cells CD4 memory resting, T cells CD4 memory activated, plasma cells, neutrophils, mast cells activated, eosinophils, and dendritic cells activated were negatively correlated with the NRS-score. The above results suggested that NRS-score plays a critical role in immune microenvironment infiltration and may have significant clinical value in CC patients.

### Correlation of NRS-Score With Tumor Mutation Burden

**Figure 9:**
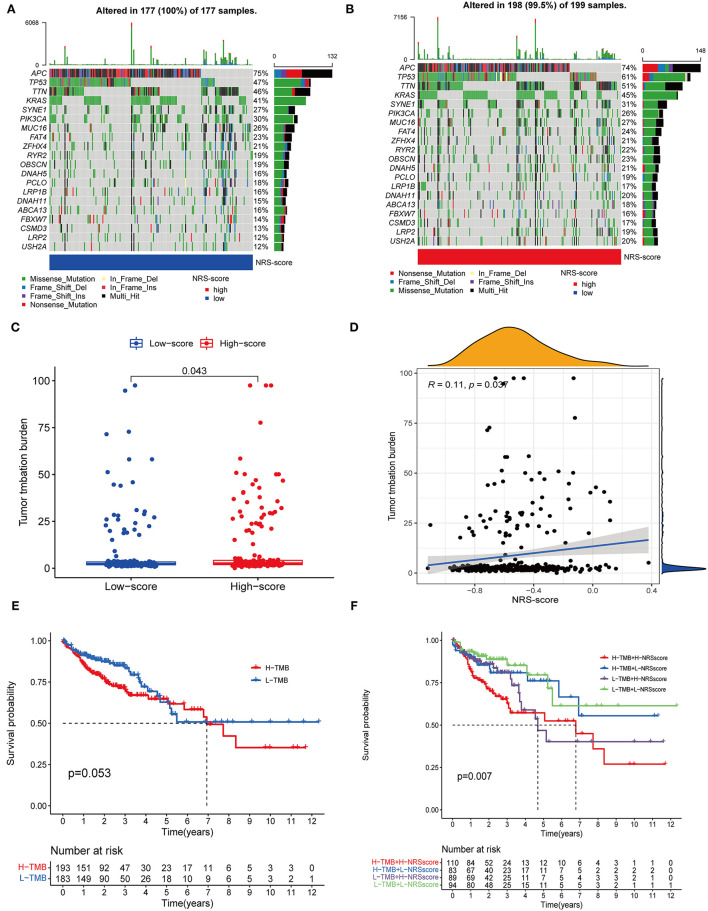
Correlation between NRS-score and somatic mutations. **(A,B)** Waterfall plots showed the top 20 frequently mutated genes in the **(A)** low NRS-score and **(B)** high NRS-score groups in the TCGA cohort. **(C)** TMB differences between the high NRS-score and low NRS-score groups. **(D)** The scatter plot depicted a positive correlation between NRS-score and TMB. **(E)** Kaplan-Meier curves for the high TMB and low TMB groups in the TCGA cohort. **(F)** Kaplan-Meier curves for patients stratified by NRS-score and TMB in the TCGA cohort.

Accumulating evidence suggests that high TMB status is associated with clinical response to immunotherapy in a variety of tumors (34, 35). Therefore, we evaluated the distribution of somatic genomic mutations between different NRS-score groups in the TCGA-CORD cohort (Figures 9A,B). The top 20 genes with the highest mutation rates in both scoring subgroups were identified, and all of these genes had mutation rates above 10%. Previous studies have shown that KRAS mutation can act as a biomarker of EGFR-targeted monoclonal antibody resistance in colon cancer patients and is associated with metastasis and poor prognosis (36, 37). In our study, KRAS mutation frequency ranked fourth in both high and low NRS-score groups. And KRAS mutation frequency was 4% higher in the high-score group than in the low-score group (45 vs. 41%), which may explain its poorer prognosis. TMB quantitative analysis confirmed a significant difference in TMB distribution among the distinct NRS-score groups (*p* = 0.043, Figure 9C). The NRS-score and TMB also showed a positive correlation (*R* = 0.11, *p* = 0.037, Figure 9D). Subsequently, we determined the optimal cut-off value of TMB (2.394) by using the minimum *p*-value method and divided the patients into high TMB group (*n* = 193) and low TMB group (*n* = 183). The results showed no significant difference in the survival prognosis of patients in the high and low TMB groups (Figure 9E). We further evaluated the synergistic effect of TMB and NRS-score subgroups in prognostic stratification. Stratified survival analysis showed that TMB status did not affect the prognosis estimates based on the NRS-score group (Figure 9F). Overall, these results suggested that the NRS-score may be potentially valuable in predicting response to immunotherapy, but its predictive potential in CC prognosis was not related to TMB.

### Correlation Between NRS-Score and Immune Checkpoint Blockade Genes

**Figure 10:**
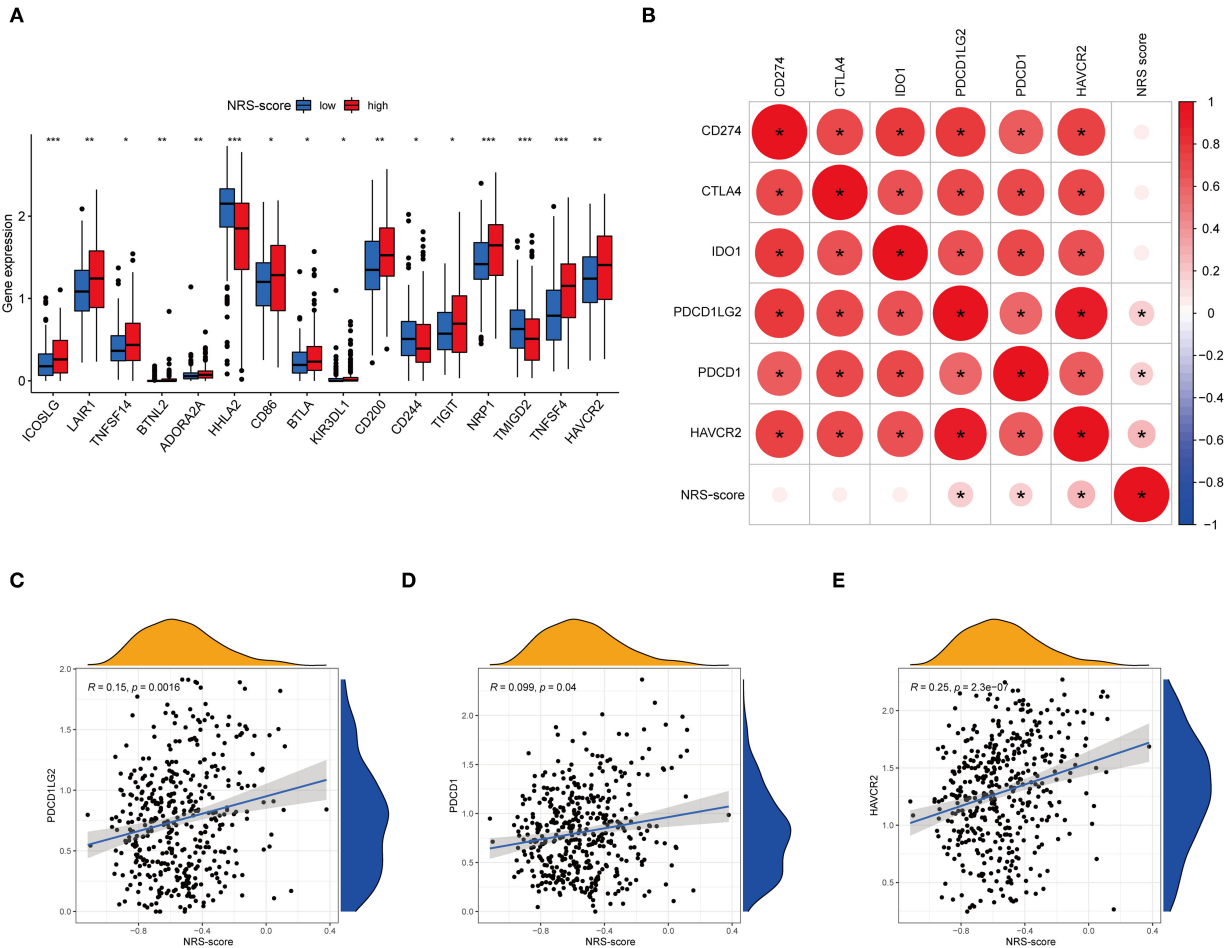
Analysis of the correlation between NRS-score based prognostic signature and the key immune checkpoint genes. **(A)** Differential expression levels of the immune checkpoint resistance-related genes in the low NRS-score and high NRS-score groups (*P < 0.05, **P < 0.01, and ***P < 0.001). **(B)** Association between immune checkpoint inhibitors CD274, PDCD1, CTLA4, PDCD1LG2, HAVCR2, and IDO1 with NRS-score. **(C)** Positive correlation between NRS-score and PDCD1LG2. **(D)** Positive correlation between NRS-score and PDCD1. **(E)** Positive correlation between NRS-score and HAVCR2.

Immune checkpoint expression has become a biomarker for the selection of immunotherapy in colon cancer patients, so we investigated the differences in expression of immune checkpoint-related genes among high and low NRS-score groups. The analysis results showed that 16 of the 47 immune checkpoint genes were significantly different in the high and low scoring groups. The immune checkpoint genes with a *p* < 0.05 are aggregated in Figure 10A. Except for HHLA2, CD244, and TMIGD2, the remaining 13 immune checkpoints were highly expressed in the high-scoring group. Subsequently, we associated six key immune checkpoint inhibitor genes, consisting of PDCD1, CD274, PDCD1LG2, CTLA-4, HAVCR2, and IDO1 (Figure 10B) (38–40). The correlation between key ICB targets and NRS-score was analyzed to reveal the potential role of NRS-score in the treatment of CC with ICB. The results showed that three of the six key immune checkpoint genes, including PD-L2 (*R* = 0.15, *p* = 0.0016, Figure 10C), PD-L1 (*R* = 0.099, *p* = 0.04, Figure 10D), and HAVCR2 (*R* = 0.25, *p* < 0.001, Figure 10E), were significantly and positively correlated with the NRS-score. The above results implied that we could select the appropriate immune checkpoint inhibitors for CC patients regrouped according to the NRS-score pattern.

### Drug Susceptibility Analyses

**Figure 11:**
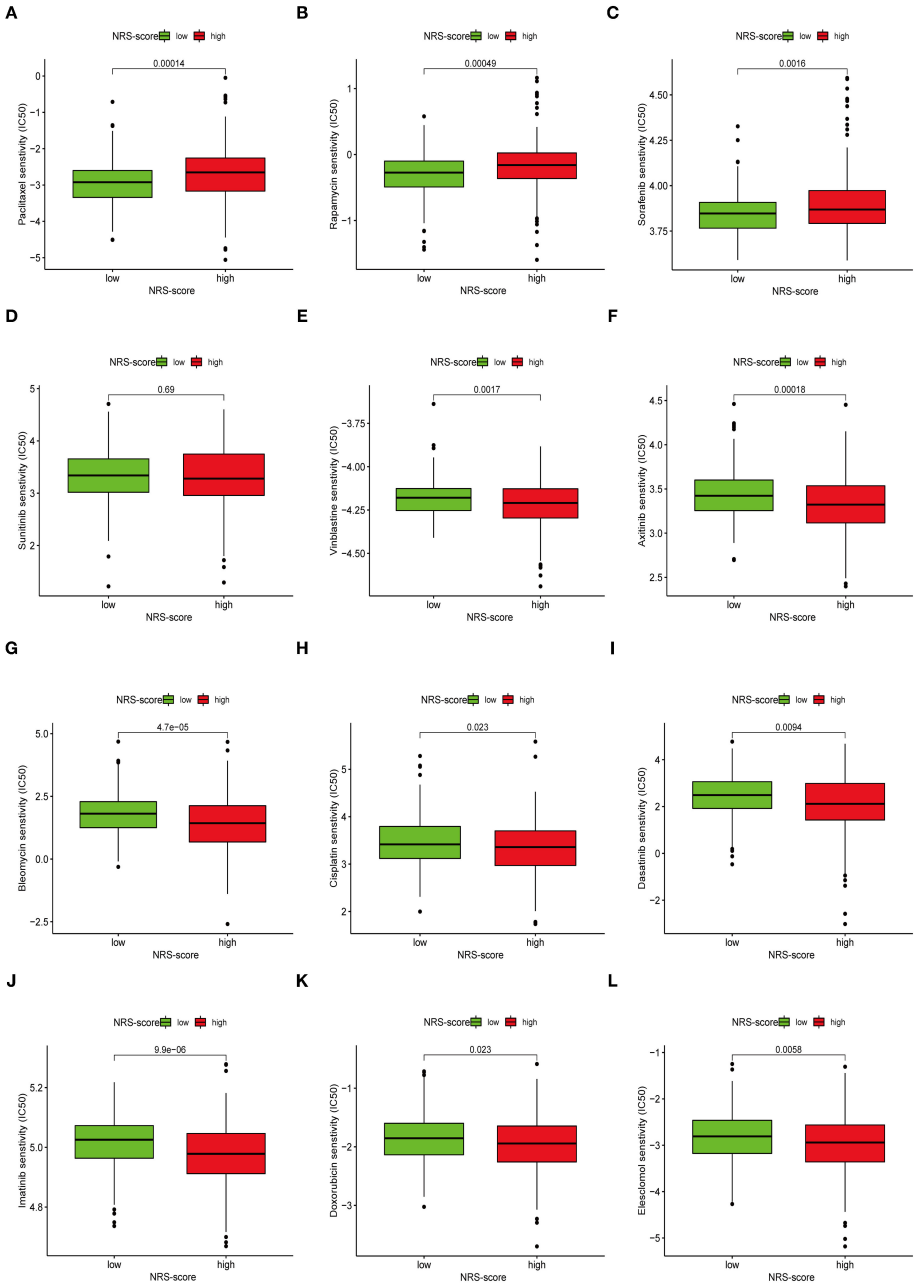
Assessment of chemotherapeutic efficacy in patients from different NRS-score groups. The sensitivity to chemotherapeutic drugs was represented by the half-maximal inhibitory concentration (IC50) of chemotherapeutic drugs. **(A)** Paclitaxel, **(B)** Rapamycin, **(C)** Sorafenib, **(D)** Sunitinib, **(E)** Vinblastine, **(F)** Axitinib, **(G)** Bleomycin, **(H)** Cisplatin, **(I)** Dasatinib, **(J)** Imatinib, **(K)** Doxorubicin, **(L)** Elesclomol.

Apart from immune checkpoint blockade therapy, we also tried to explore the relationship between NRS-score and the efficacy of common chemotherapeutic and targeted therapeutics agents in colon cancer patients in the TCGA-CORD project (Figure 11). The results found that patients in the low NRS-score group were associated with lower IC50 of the anti-cancer drugs paclitaxel, rapamycin, and sorafenib. In contrast, patients in the high NRS-score group were associated with lower IC50 of vinblastine, axitinib, bleomycin, cisplatin, doxorubicin, imatinib, dasatinib, and elesclomol. The above results suggested that NRS-score could be used as a potential predictor of anti-cancer drug treatment sensitivity.

## Discussion

Necroptosis has been found to play distinct roles in various types of tumors, with a tumor growth-promoting role in glioblastoma, pancreatic cancer, and lung cancer (41–43), a tumor growth-inhibiting role in colorectal cancer, gastric cancer, head and neck squamous cell carcinoma, and melanoma (44–47), and a bi-directional role in breast cancer (48, 49). Therefore, we cannot evaluate the prognostic value of CC based solely on the expression of individual necroptosis regulators. Targeting necroptosis is considered an effective way to overcome chemoresistance, making it a new approach for cancer treatment (17). A recent study constructed a necroptosis-related LncRNA prognostic model to distinguish hot and cold tumors in gastric cancer, thereby predicting patient prognosis and immunotherapy response (50). However, the relationship between CC and necroptosis is unclear. Therefore, this study investigated the integrated role of necroptosis-related genes in the CC phenotype and TME. In addition, a signature related to necroptosis was constructed to predict the molecular subtype of necroptosis, prognosis, immunotherapy, and the effect of chemotherapy in CC.

Herein, we first comprehensively evaluated the expression profile and genetic variation landscape of necroptosis-related genes in TCGA-COAD patients. We found that 62 of 76 necroptosis genes were differentially expressed in cancer and normal tissues. At the genetic level, 190 of 399 patients were found to have undergone mutations with mutation frequencies ranging from 1 to 15%, with BRAF having the highest mutation of all necroptosis regulators. Studies have reported that BRAF mutations and KRAS mutations are independent and exclusive of each other, account for 5–15% of metastatic colon cancers, and are associated with poor prognosis in advanced colon cancer (51). The above results suggested that dysregulation of necroptosis regulators may be closely related to the high heterogeneity of colon cancer. Next, we classified the CC patients in the meta-dataset according to the expression of differentially expressed necroptosis-related regulators. The C2 subtype had a better survival outcome compared to the C1 subtype. ssGSEA analysis showed significant differences in immune cell infiltration between the two molecular subtypes. GSVA analysis showed that the C2 subtype was mainly enriched in metabolism and repair activation-related pathways, while the C1 subtype, with a poorer prognosis, was mainly enriched in oncogenic and stromal activation-related pathways. Our results revealed that the two necroptosis subtypes have completely different clinical outcomes and TME infiltration characteristics due to various heterogeneities. We further performed enrichment analysis based on differential genes between the two molecular subtypes, and the results showed that DEGs are involved in various immune-related functions and pathways, such as chemokine receptor binding, Cytokine-cytokine receptor interaction, IL −17 signaling pathway, and TNF signaling pathway.

To accurately predict the prognosis of individual CC patients, we constructed a prognostic gene signature based on these phenotype-related differential genes, which was named NRS-score. Survival analysis showed that patients in the low NRS-score group had significantly prolonged OS compared to those in the high NRS-score group, as confirmed by the independent GEO cohort (GSE39582). Multivariate Cox regression analysis showed that NRS-score, T-stage, N-stage, and M-stage were identified as independent prognostic factors and were included in the nomogram. The calibration curves indicated that the nomogram was an effective tool for predicting the prognosis of CC. Our results demonstrated that the constructed model could well-differentiate colon cancer patients and predict prognosis, thus helping to develop individualized treatment plans based on the NRS-score of patients.

The prognostic signature we constructed contains a total of eight hub genes, which have attracted much attention in the cancer field so far. Gastrin-releasing peptide (GRP) and its receptor (GRPR) have been reported to be aberrantly expressed in the colon after malignant transformation and have been associated with delayed tumor recurrence and improved survival (52). They may retard tumor progression by enhancing the attachment of CC cells to the extracellular matrix and promote natural killer lymphocyte binding leading to tumor cell solubilization. Matrix metalloproteinases (MMPs) that contain multiple zinc-dependent endopeptidases are involved in tumor progression, angiogenesis, and immune escape (53). Studies have shown that high expression of MMP-1 is associated with local and distant metastasis in colon cancer (54, 55). Serum levels of MMP10 are also an independent poor prognostic marker in patients with colon cancer (53). Serine protease inhibitor, Kazal type 1 (SPINK1), a trypsin inhibitor, is closely associated with inflammatory states and the proliferation and metastasis of various cancer cells (56). Silencing SPINK1 significantly reduces proliferation and invasion of CC cells and leads to upregulation of various metallothioneins, which increases sensitivity to chemotherapeutic agents (57). However, in another study, high SPINK1 expression was associated with a good prognosis in patients with advanced colorectal cancer receiving targeted cetuximab-based therapy (58). Calcium-activated chloride channel (CLCA) modulators are involved in many biological processes, including apoptosis, airway inflammation, and immune regulation (59, 60). Wei et al. analyzed CLCA4 expression in colorectal normal, adenoma, and cancer tissues based on immunohistochemical tissue microarrays and found a gradual decrease in tumor progression (60). In addition, low CLCA4 expression was associated with overall survival in patients with various tumors, including CC, head and neck cancer, gastric cancer, and breast cancer. Lysosomal-associated membrane protein family member 5 (LAMP5) is a member of the lysosomal-associated membrane protein family. Shi et al. found that LAMP5 is an immune marker associated with colon cancer prognosis and tumor-infiltrating lymphocytes (61). Fatty acid binding protein 4 (FABP4) is an intracellular lipid transport protein that can bind and transport hydrophobic fatty acids (62). Numerous studies have shown that FABP4 is a poor prognostic factor in metastatic cancers such as colon (63), cervical (64) and breast (65) cancers. G protein coupled receptor-associated sorting protein 1 (GPRASP1) has been reported to be aberrantly expressed in several cancer types, including liver, breast, brain, and lung cancers, and may serve as a potential tumor biomarker for patients (66). However, its role in colon cancer has not been reported.

The regulation of the inflammatory reactions induced by necroptosis is crucial in the tumor microenvironment. The present study showed that different NRS-score groups exhibited different TME infiltration characteristics. NRS-score showed a significant positive correlation with ESTIMATES score and stromal score, but a negative correlation with tumor purity. In other words, tumors in the high NRS-score group were of lower purity and had a worse prognosis, consistent with the findings of several previous studies in colon cancer (67, 68). We hypothesized that this might be due to the recruitment of more immune-suppressive cells in low-purity tumors than in high-purity tumors. Therefore, we further investigated the relationship between NRS-score and the abundance of infiltrating immune cells. We found significant differences in immune cell infiltration fraction between the two groups, particularly in the activation of T cell CD4 memory, macrophages, neutrophils, and dendritic cells. A previous study demonstrated that in TME, macrophages are in an immunosuppressed state and promote tumor development by preventing T cells from eliminating cancer cells (69). Necroptosis could protect tumors against intrinsic anti-tumor immune responses by creating an immune-suppressive microenvironment, which might explain the poor outcome of patients in the high NRS-score group.

Recently, targeted immune checkpoint blockade (ICB) therapy has been considered as a promising approach for the treatment of CC (70). However, only about 5% of patients with progressive dMMR/MSI-H CC are more sensitive to immune checkpoint inhibitors. Currently, TMB is considered to predict ICB treatment efficacy and has become a valuable biomarker in various cancer types, with high TMB often corresponding to better immunotherapy efficacy (71, 72). We found that the proportion of somatic mutations differed significantly between NRS-score groups and that NRS-score was significantly positively correlated with TMB. This means that the NRS-score may reflect the response to immunotherapy to some extent. Previous studies have shown that the expression of immune checkpoint molecules is an important factor in tumor immune escape (73). Immune checkpoint molecules can play a key role in tumor progression by promoting tumor cell evasion from anti-tumor immunity (74). Our analysis showed that 13 of the 16 differentially expressed immune checkpoint genes were overexpressed in the high NRS-score group. Also, NRS-score was significantly and positively correlated with three of the six ICB key targets (PD-L2, PD-L1, and HAVCR2). These results indicated that tumor cells in the high NRS-score group protected themselves from attack by expressing immune checkpoint molecules. Also, the expression of immune checkpoint molecules can be used as a predictor of immunotherapy response. Thus, a prognostic signature based on necroptosis may provide new insights into the prediction of ICB treatment outcome in CC.

Finally, we determined the drug sensitivity of different anticancer drugs in the treatment of patients with CC in distinct NRS-score groups. Based on IC50 values, vinblastine, axitinib, bleomycin, cisplatin, doxorubicin, imatinib, dasatinib, and elesclomol showed better responses in the high NRS-score group, while paclitaxel, rapamycin, and sorafenib in the low-score group showed better response. Screening chemotherapeutic agents according to the molecular subtypes of patients for individualized treatment is the inevitable way to overcome drug resistance.

In summary, our study was designed to identify molecular subtypes of necroptosis in CC patients, construct a prognostic signature based on genes differentially expressed between subtypes, and link necroptosis to patient prognosis and immune microenvironment. The NRS-score based on the prognostic signature can predict not only the long-term survival of patients but also their clinical response to anti-PDL1 immunotherapy, the efficiency of chemotherapy and targeted therapy. However, there are still some limitations of our study. First, our study samples were all based on retrospective data, and large-scale prospective data are needed to validate our findings. Second, the specific molecular mechanisms of the eight prognostic gene signatures in colon cancer require further *in vivo* and *in vitro* experiments to investigate. In addition, the clinical application of NRS-score in the individualized treatment of colon cancer needs to be further investigated. These not only raise the challenges but also give us more incentives to keep digging.

## Data Availability Statement

The datasets presented in this study can be found in online repositories. The names of the repository/repositories and accession number(s) can be found in the article/Supplementary Material.

## Author Contributions

RH wrote the article. MZ, LH, JH, and CM processed the data analysis. XW and YL conceived of this study. YF revised the final manuscript. All authors read and approved the final manuscript, contributed to the article, and approved the submitted version.

## Funding

This study was supported by Jiangsu Innovative Team Leading Talent Fund (CXTDC2016006 and QNRC2016446), Jiangsu 333 Talent Fund (BRA2020016), Jiangsu Provincial Key Research and Development Special Fund (BE2015666), Jiangsu Six High Peak Talent Fund (WSW-205 and WSW236), Zhenjiang Key Research and Development Fund (SH2021038), and Suqian Science and Technology Support Project Fund (K201907).

## Conflict of Interest

The authors declare that the research was conducted in the absence of any commercial or financial relationships that could be construed as a potential conflict of interest.

## Publisher's Note

All claims expressed in this article are solely those of the authors and do not necessarily represent those of their affiliated organizations, or those of the publisher, the editors and the reviewers. Any product that may be evaluated in this article, or claim that may be made by its manufacturer, is not guaranteed or endorsed by the publisher.
