# Supplementary material for: Integrated Analysis of Necroptosis-Related Genes for Prognosis, Immune Microenvironment Infiltration, and Drug Sensitivity in Colon Cancer
Source: Front Med (Lausanne). 2022 Apr 11;9:845271. doi: 10.3389/fmed.2022.845271 (PMC9036446; doi:10.3389/fmed.2022.845271)
Supplement: Supplementary file 1 [file Data_Sheet_1.pdf]

Supplementary Figures

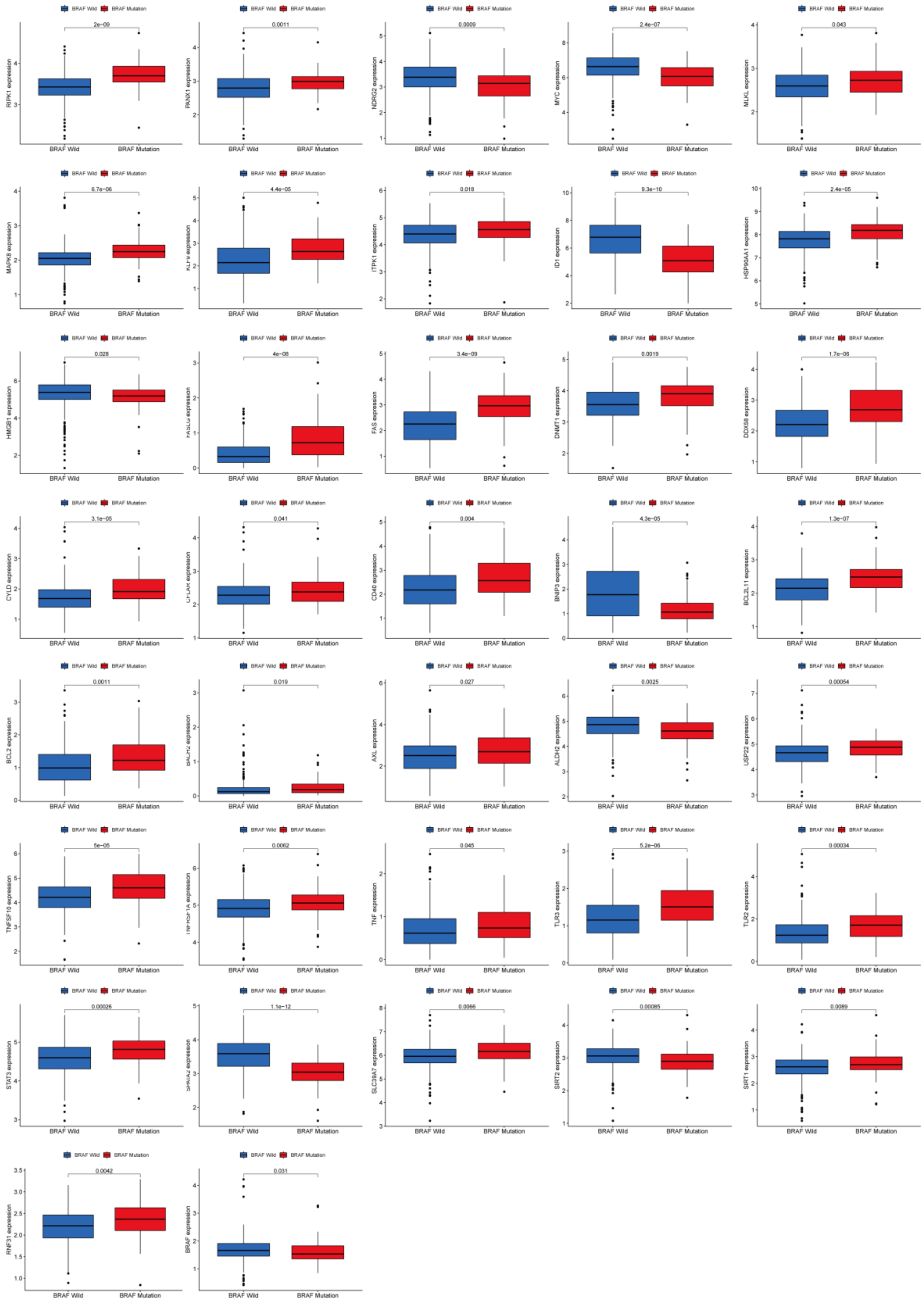

Figure S1. The relationship between BRAF mutation and expression level of

necroptosis-related genes in colon cancer.

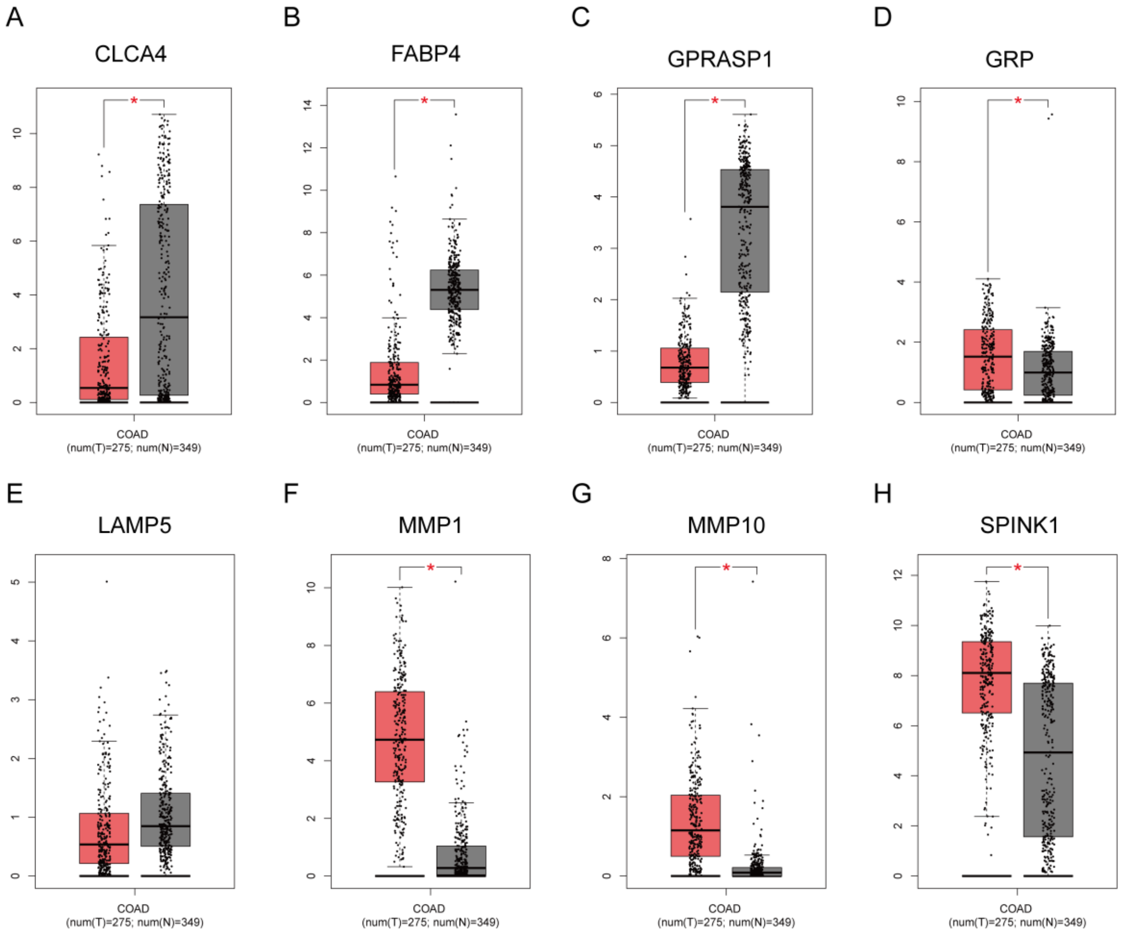

Figure S2. (A-H) Differential expression of the 8 model gene in the Gene Expression Profiling Interactive Analysis (GEPIA) online databases .

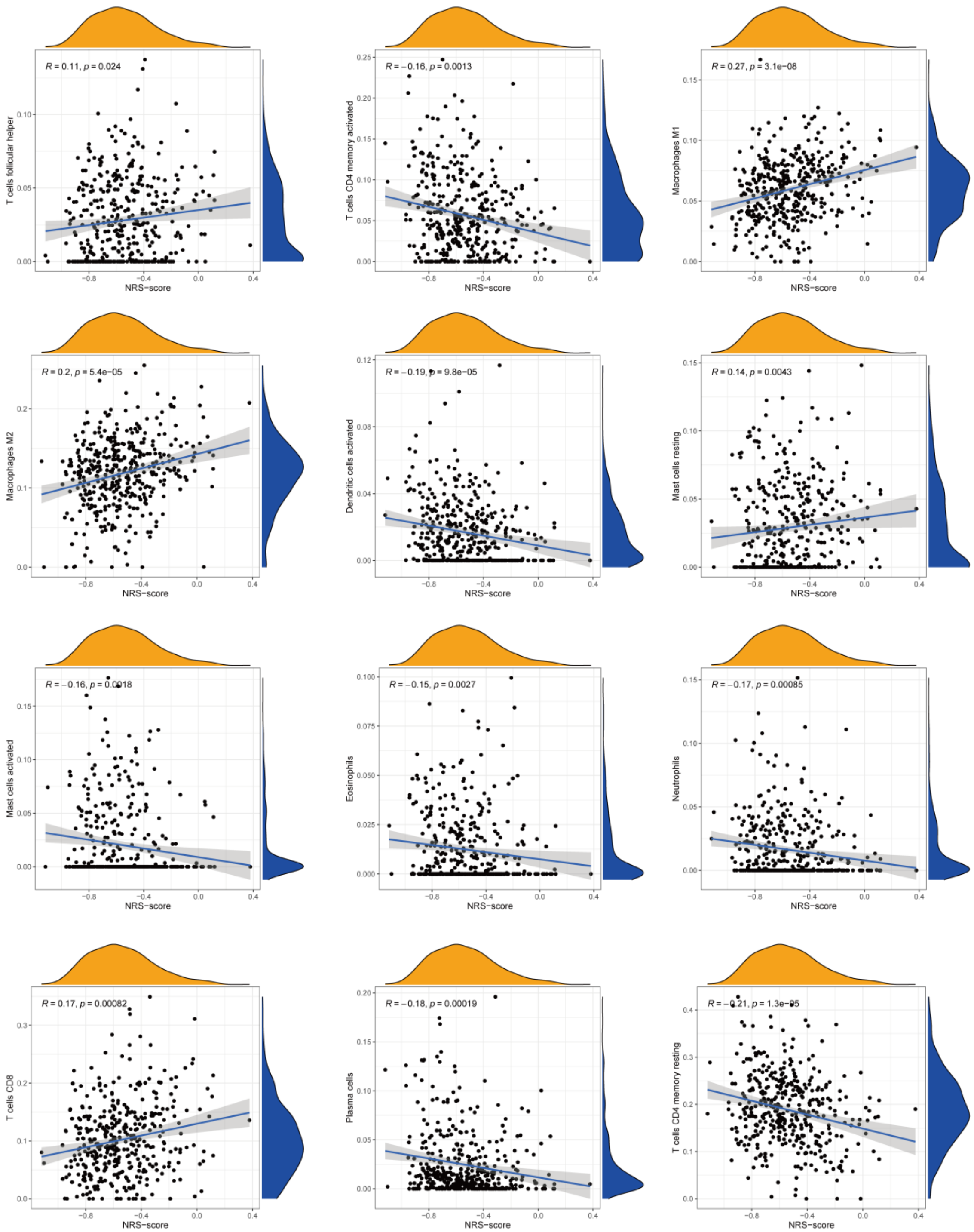

Figure S3. Correlation test between immune cells and NRS-score, only TICs with significant correlation are plotted. The blue line in the plot is the fitted linear model, and the shading around it represents the 95% confidence interval.
